# Rashtriya Bal Swasthya Karyakram: An Approach to Public Health Plastic Surgery

**DOI:** 10.1055/s-0046-1827059

**Published:** 2026-08-12

**Authors:** Veena Kumari Singh, Maneesh Singhal

**Affiliations:** 1 Department of Burns & Plastic Surgery, All India Institute of Medical Sciences (AIIMS), Patna, Bihar; 2 Department of Plastic Surgery, All India Institute of Medical Sciences (AIIMS), New Delhi, India

Since its origin in India, plastic surgery has evolved considerably over time, with newer reconstructive strategies for various anatomical regions. However, pediatric reconstructive surgery is yet to be delivered with an optimum balance between demand and supply, especially in rural and underserved regions. Rashtriya Bal Swasthya Karyakram (RBSK) is a government scheme focused on the treatment of infants and children born with congenital anomalies. The scheme has been adopted by the majority of the state health machinery; however, despite it, the plastic surgeons are yet to become fully aware of the RBSK scheme's potential to become one of the largest referral platforms for reconstructive plastic surgery in India.

## RBSK Scheme

In 2013, the Ministry of Health and Family Welfare (MoHFW) launched the RBSK scheme in all states and union territories under the National Health Mission for children in the age group 0 to 18 years with the objective of early identification and management for 32 selected health conditions under 4D's—Defects at birth, Developmental delays, Diseases, and Deficiencies. 1 The birth defects requiring plastic surgery intervention included in this scheme are cleft lip, palate, craniofacial clefts, microtia, hand anomalies, syndactyly, polydactyly, vascular anomalies, congenital ptosis, and other reconstructive conditions. District early intervention centers (DEIC) are set up for the screening of defects based on physical examination, issuing of RBSK pre-authorization certificate, and referral to various government institutions covered under this scheme.

A recent hospital-based study on birth defects among children enrolled in the RBSK scheme at a tertiary care center showed that cleft lip, palate, and other plastic surgery congenital defects constitute a larger proportion, second only to neural tube defects. 2 Another study revealed that RBSK is already benefiting the cleft patients, implying that this scheme is equally effective as Smile Train and other supporting agencies as far as screening quality, referral pathways, and multidisciplinary coordination are concerned. 3

## RBSK—A Platform for Public Health Plastic Surgery

From a public health perspective, RBSK, being a national government initiative, provides an existing link between mobile health teams, DEICs, and tertiary referral hospitals. This allows screening, referral, evaluation, and treatment to occur within a defined system of care. Also, as a community-based scheme involving the Anganwadi centers, schools, and village-level health services, children with congenital anomalies can be identified early and referred for further evaluation. This is particularly relevant in rural and underserved areas, where parents may not recognize the condition or may delay seeking specialist care because of financial, social, or geographical barriers. Moreover, the RBSK scheme offers free-of-cost treatment to patients, thereby reducing the out-of-pocket expenditure and financial burden and making the family more compliant. Due to the effective implementation of this scheme at various government centers, robust epidemiological data on congenital anomalies is being generated, which is required for measuring the impact of the program, policy making, and evaluating long-term surgical outcomes. 4

Despite these strengths, the identification of congenital anomalies requiring plastic surgical intervention remains limited. Mobile health teams and DEIC personnel are generally more familiar with cleft lip and palate, while other congenital conditions may not always be recognized or referred to at the appropriate time. One of the reasons for this may be the limited involvement of plastic surgeons in the planning and implementation of the program. Specialties such as pediatric surgery, ophthalmology, otorhinolaryngology, and dentistry have been given greater emphasis in the existing operational framework, whereas plastic surgery is not represented uniformly. This is important because delayed diagnosis and referral may result in treatment at a later stage, increased reconstructive difficulty, and poorer functional or aesthetic outcomes.

The time has come for the plastic surgeons to take the lead through various measures, such as in developing simple RBSK screening manuals, training frontline health workers, creating pictorial referral guides, supporting teleconsultation services, and strengthening DEIC teams to identify cases that require early referral. Plastic surgeons at government medical colleges must take efforts in facilitating the MOU between the state health department and their college/institutes for getting it designated as RBSK tertiary care centers. Appointing a nodal officer from the department can help in coordinating evaluation, pre-authorization, multidisciplinary care, surgery, and follow-up.

The current situation also demands greater engagement between the Association of Plastic Surgeons of India (APSI) and the National Health Mission. As a policy-making organization and guiding the code of conduct for plastic surgeons, APSI should volunteer for updating the list of plastic surgery congenital anomalies under RBSK, developing standard referral and treatment protocols, and defining the minimum requirements for participating centers. It can also help in developing a training program for healthcare workers involved in child screening. From a plastic surgery perspective, the RBSK scheme should be looked upon as a cornerstone of public health plastic surgery in India. Greater involvement of plastic surgeons, structured referral pathways, appropriate funding, multidisciplinary support, and systematic follow-up could improve access to timely reconstructive care and reduce long-term disability among affected children. Such participation would also strengthen the contribution of plastic surgery to child health and the broader goals of Viksit Bharat 2047.
